# Extraction of Bacterial RNA from Soil: Challenges and Solutions

**DOI:** 10.1264/jsme2.ME11304

**Published:** 2012-02-22

**Authors:** Yong Wang, Masahito Hayatsu, Takeshi Fujii

**Affiliations:** 1 Environmental Biofunction Division, National Institute for Agro-Environmental Sciences, 3–1–3 Kannondai, Tsukuba, Ibaraki 305–8604, Japan

**Keywords:** soil, humic acids, RNA extraction, gene expression, metatranscriptomics

## Abstract

Detection of bacterial gene expression in soil emerged in the early 1990s and provided information on bacterial responses in their original soil environments. As a key procedure in the detection, extraction of bacterial RNA from soil has attracted much interest, and many methods of soil RNA extraction have been reported in the past 20 years. In addition to various RT-PCR-based technologies, new technologies for gene expression analysis, such as microarrays and high-throughput sequencing technologies, have recently been applied to examine bacterial gene expression in soil. These technologies are driving improvements in RNA extraction protocols. In this mini-review, progress in the extraction of bacterial RNA from soil is summarized with emphasis on the major difficulties in the development of methodologies and corresponding strategies to overcome them.

Many tales about the Earth can be found in the cultures of ancient China, ancient Greece, and other nations throughout the world. Although we live on the Earth, it remains mysterious to us. Most of our food originates from soil, which forms a very thin layer on the surface of the Earth. To understand the Earth better, knowledge of soil and the microorganisms living in it should be obtained. During the last century, one of the major achievements of soil microbiologists was isolating bacterial strains from soil and surveying their population in soil environments using culture-based methods; however, the great number of bacterial species (35, 100, 125, 126) in soil makes the isolation and identification of new bacterial species a never-ending task. Although much effort has been devoted to the development of new strategies to isolate new species from soil (48, 78, 114, 118, 133), many bacterial species are resistant to culture. Because approximately 99% of bacteria in soil remain unidentified and/or are difficult to culture (124), culture-based methods have limitations for the survey of bacterial populations in soil. These limitations have motivated researchers to search for breakthrough culture-independent approaches. After it was approved for use in a wide range of life science applications (8, 22, 70, 89, 135), the polymerase chain reaction (PCR) technique, which appeared in the mid-1980s (82), was used by soil microbiologists soon after its introduction to detect bacterial genes in soil (15, 50, 94, 104). With the increasing use of PCR, more soil microbiological studies focused on specific genes in soil bacteria, mainly the small subunit ribosomal RNA gene (11, 44, 65, 103, 120). Culture-independent molecular techniques have proven that the microbial world is genetically and functionally more complex and diverse than previously hypothesized on the basis of culture-dependent studies (88, 148). Culture-independent methods provide us with large amounts of information about bacterial species in soil, and this information is useful for identifying newly isolated bacterial species and surveying the bacterial community in soil environments (57, 71). Internet databases, such as the Ribosomal Database Project (RDP) (http://rdp.cme.msu.edu/), 16SpathDB (146), and Greengenes (24), facilitate the dissemination of new information to soil researchers.

Because PCR techniques require DNA as a template, many researchers have used bacterial genomic DNA extracted from soil as templates for PCR detection of bacterial genes in soil (11, 65, 103). DNA only provides us with information about the existence of bacteria in soil; it cannot provide us with information about gene expression, which is important to understand bacterial activities in soil, such as bacterial growth, degradation activities of various compounds, and bacterial responses to environmental factors. For this reason, a study using reverse transcription-polymerase chain reaction (RT-PCR) to detect bacterial gene expression in soil was launched in the early 1990s (44, 105, 128). Recently, the cDNA clone library was also used to investigate active genes in soil (10, 108). Because both RT-PCR and the cDNA clone library require bacterial RNA as a template for converting RNA into cDNA, direct extraction of bacterial RNA from soil is a key procedure in both techniques and is of great interest. In the past 20 years, many methods of RNA extraction from soil have been reported (3, 9, 29, 43, 44, 54, 68, 75, 76, 80, 92, 105, 106, 128, 138, 139); however, until now, there has been no method for RNA extraction from all types of soil, so researchers had to choose or develop soil RNA extraction methods to fit their own research purposes. The lack of a universal RNA extraction method for all soils hindered the study of bacterial gene expression in soil. Recently, the application of RNA extracted from soil has been extended to microarray (74, 142) and high-throughput sequencing (62, 130) analysis, which are more powerful than RT-PCR and may provide us with information about the global gene expression of soil bacteria.

This review primarily discusses the major difficulties encountered in extracting RNA from soil and corresponding strategies to overcome those difficulties, rather than each detailed procedure in the protocol.

## Overview of RNA extraction from soil

RNA extraction from soil can be divided into three stages: cell lysis, extraction of RNA from the soil matrix, and purification of RNA. At the cell lysis stage, bead beating has become popular over the past 10 years (3, 43, 68, 76, 92, 106, 138, 139), although several other methods, such as sonication (44), grinding after freezing in liquid nitrogen (54), and enzymatic lysis by lysozymes (80), have also been used. To protect RNA from degradation by RNase, inactivation reagents for RNase, such as guanidine thiocyanate, guanidine isothiocyanate, 2-mercaptoethanol, or dithiothreitol are normally added to the extraction buffer so that the RNase molecules can be inactivated immediately after they are released from cells. Inactivation of RNase can also be performed prior to cell lysis, for example, the pre-treatment of soil with RNA*later* (Ambion, Austin, USA) (76). After cell lysis, RNA molecules, together with DNA and proteins, are released from cells into the soil suspension; meanwhile, humic substances are also released from soil particles; therefore, the soil suspension is a mixture of many kinds of molecules, including RNA and humic substances. At the second stage, the nucleic acids can be separated from the soil matrix, proteins, and cell debris by phenol extraction. At this step, humic substances can be co-extracted together with the nucleic acids (139). Then, RNA precipitation by ethanol, isopropanol, or polyethylene glycol (PEG) is typically required to reduce the volume of the sample and to remove various salts. At the third stage, RNA samples are purified by spin columns, including gel filtration (size exclusion) (75, 80, 106, 138, 139) and ion exchange (54, 76) chromatography columns. Commercial kits for RNA extraction from soil are also available, and are summarized in Table 1. A recent report evaluated a selection of these commercial kits (25).

## Difficulties in recovering bacterial RNA from soil

### Contamination by humic substances

**Fig. 1:**
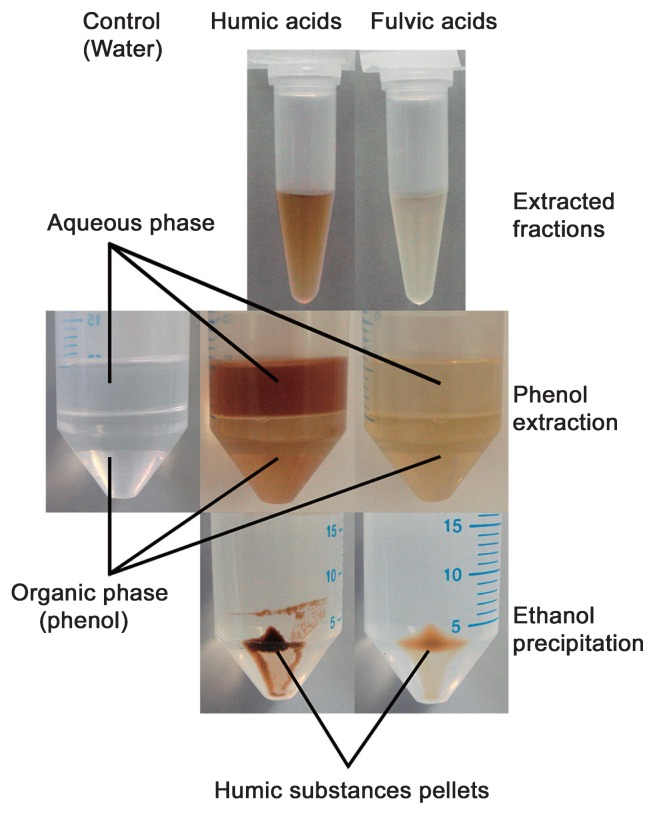
The behavior of humic and fulvic acids during phenol extraction and ethanol precipitation. Humic and fulvic acids were prepared as previously described (139). Citrate-saturated phenol at pH 4.3 was used for extraction, and water was used as a control to show the original color of the phenol reagent used. The aqueous layer was transferred to a fresh tube, followed by the addition of 0.1 volume of 3 M sodium acetate (pH 5.2) and 2 volumes of ethanol for precipitation.

Impurities are extracted from soil along with RNA, and the majority of these impurities are humic substances, which are dark-colored, heterogeneous organic compounds in soil (115). Based on their solubility under acidic or alkaline conditions, humic substances in soils can be divided into three main groups: humic acids, which are soluble under alkaline conditions but not acidic conditions; fulvic acids, which are soluble under all pH conditions; and humin, which is the insoluble fraction (115). Because humin cannot be extracted by any water solution, the predominant humic substances co-extracted with RNA should be humic and fulvic acids. Fulvic acids inhibit PCR amplification, but only at high concentrations (59). Compared with fulvic acids, the effect of humic acids on biological experiments has been well studied because they present difficulties in various molecular biological experiments. Humic acids have been shown to interfere with enzyme reactions (restriction endonuclease, DNase, and RNase) (122), PCR amplification (122, 129, 141), DNA-DNA hybridization (113, 122), transformation of competent cells (122), nucleic acid detection and measurement (4, 152), RNA hybridization (2), and RT-PCR (141). Thus, the removal of humic substances from soil RNA samples is critical to molecular analysis; however, complete removal is rather difficult (45). As shown in Fig. 1, only a fraction of humic and fulvic acids can be removed by phenol extraction, and both can be precipitated by ethanol, which is somewhat similar to DNA and RNA.

### Adsorption of RNA by soil

As mentioned above, there have been a lot of successful cases of RNA extraction from diverse soils; however, RNA extraction from Andosols is a challenge. Andosols (volcanic ash soils) can be found all over the world. In Japan, Andosols cover about 16.4% of land surface and 46.5% of arable upland fields (41); thus, it is necessary to establish a method for RNA extraction from Andosols to facilitate the study of bacterial gene expression. For this reason, we attempted RNA extraction from Andosols with a popular commercial kit, RNA PowerSoil Total RNA Isolation Kit (MO BIO, Carlsbad, CA, USA). Unfortunately, RNA extraction failed in all Andosol soil samples tested (Wang *et al.*, unpublished data), although this commercial kit has been proven to extract RNA from diverse soils successfully (1, 23, 25, 101, 142, 145). It is true that soil possesses detectable extracellular RNase activities (42); however, recent reports suggest that RNA could survive in the presence of extracellular RNase in soil (7, 30). Also, almost intact bacterial rRNA could be extracted from an Andosol with an extraction buffer amended with DNA (52). Thus, the failure of RNA extraction from Andosols is possibly caused by RNA adsorption by soil but not RNA degradation by RNase. RNA adsorbs to soil very quickly. About 50–90% of the adsorbed RNA molecules were adsorbed to clay within one hour (40), and 85% of the maximum adsorption occurred on allophane (one of the major components in Andosols) within 30 min (121). It is known that all RNA components (mononucleotides, nucleosides, bases, phosphate and ribose) and nucleotides possessing different numbers of phosphate groups can be adsorbed by soil (13, 17, 18, 40, 47, 64). Also, all of the RNA components could be adsorbed by allophane (46). Although both DNA and RNA could be adsorbed by soil (40), it seems that RNA is more difficult to extract from soil than DNA. First, the ribose in RNA has one more hydroxyl group than the 2-deoxyribose in DNA. This hydroxyl group may result in stronger adsorption of RNA on soil than that of DNA since ribose hydroxyl groups are involved in the binding of ribose with soil (17). Second, the free extracyclic functional groups on the bases in the single-strand structure of RNA (partial base pairing may occur in some regions of RNA molecules) could form hydrogen bonds with soil surface (99), which may also result in stronger adsorption of RNA on soil than that of DNA. This is supported by a previous report in which, from the same Andosol, DNA was successfully extracted by a skim milk amended extraction buffer, whereas RNA failed to be extracted using the same buffer (52). A recent report revealed that soil clay content significantly affects RNA isolation yields and that quantitative RT-PCR (qRT-PCR) analysis and all RNA isolation methods tested in that study were negatively affected by high clay soils (86); however, it is unclear whether the clay content in Andosols is the major factor affecting the adsorption of RNA by Andosols. Therefore, efforts are still required to investigate the mechanism of RNA adsorption by Andosols.

### Existence of rRNA in the total RNA of soil bacteria

Unlike eukaryotic mRNA, bacterial mRNA does not normally possess a poly(A) tail, which makes the purification of bacterial mRNA difficult. Although recent studies revealed that some mRNA molecules in bacteria possess a poly(A) tail, those mRNA molecules only occupy a small portion of the whole transcriptome and will be subject to fast degradation (26); thus, obtaining bacterial mRNA without the contamination of rRNA is a challenge. For RT-PCR and qRT-PCR, the existence of rRNA in an RNA sample is not a problem because of the usage of specific primers. Signal saturation caused by rRNA can also be avoided at the array design step in microarray analysis by excluding the probes for rRNA. However, in a transcriptomic study using high-throughput sequencing technologies, such as Roche 454, Illumina, and ABI SOLiD, information on functional genes in a high-throughput sequencing dataset is rather limited because rRNA molecules are dominant in the total bacterial RNA (130); thus, the removal of rRNA from total RNA is essential before deep sequencing.

### Low yield of RNA from soil

It is easy to recover large quantities of total RNA from a pure culture of bacteria (tens of micrograms per extraction), but it is rather difficult in soil. According to reports, the yields of RNA extracted from soil range from tens of nanograms to several micrograms per gram of soil (3, 9, 75, 76, 80, 106, 138, 139). Such a wide range of RNA yield may be caused in many ways, such as by the amount of living microorganisms in soil samples, contamination of humic substances or the loss of RNA during purification. The quantity of RNA extracted with all of the previously mentioned methods could be sufficient for RT-PCR and qRT-PCR analysis, in which ten (one-step qRT-PCR) to several hundred (two-step qRT-PCR) nanograms of total RNA may be sufficient; however, microarray and high-throughput sequencing analysis require microgram levels of RNA, especially for the detection of rare sequences. To collect a sufficient quantity of RNA for microarray or high-throughput sequencing analysis, RNA extraction from a large amount of soil is required. It has been reported that increased amounts of soil for RNA extraction resulted in the accumulation of humic substances in RNA samples (139). To obtain high-purity RNA, more purification procedures are required. Thus, an RNA extraction method developed for microarray analysis has more purification procedures than those for RT-PCR or qRT-PCR analysis and normally has a low RNA yield because of the loss of RNA during purification (139).

## Strategies to overcome these difficulties

### Removal of humic substances

Many methods have been tested or used to remove humic substances from RNA extracted from soil, including chemical flocculation with Al_2_(SO_4_)_3_ under alkaline conditions prior to cell disruption (92), control of cell disruption conditions (temperature and pH) (139), addition of cetyltrimethyl ammonium bromide (CTAB) to the extraction buffer (3, 43), precipitation of RNA by PEG (3, 43), adsorption by polyvinylpolypyrrolidone (PVPP) (75), co-precipitation with guanidine hydrochloride (44), and chromatography using gel filtration (75, 80, 106, 138, 139) and ion exchange (54, 76, 138) columns. It has been proven that DNA irreversibly binds to humic acids under acidic conditions (19); this probably also occurs with RNA. Thus, when substantial loss of RNA is expected because of large amounts of humic substances released into the extraction solution, the complete removal of humic substances prior to cell disruption is highly recommended (93). The half-life of bacterial mRNA is very short, ranging from no more than 30 s to more than 20 min (28), and thus, unlike DNA extraction, a pre-wash step is inappropriate for RNA extraction from soil (140). For this reason, the control of cell disruption conditions, such as the temperature or pH of the extraction buffer (139), or using an extraction buffer amended with CTAB (3, 43) can be more helpful than other methods of controlling the release of humic substances into the aqueous phase. Phenol extraction is a common procedure to remove proteins from the cell lysate. As shown in Fig. 1, to some extent, phenol extraction also removes humic and fulvic acids. Precipitation of RNA is normally required before a purification procedure to reduce the volume of the RNA sample and to remove various salts and partial humic substances. Although ethanol is commonly used, isopropanol and PEG show higher recoveries of nucleic acids with low contamination of humic acids (20). In most cases, one or multiple purification procedures are required to remove humic substances completely. Because the weight average molecular weight of humic and fulvic acids in soil is less than 20 kDa (91), which is slightly lower than typical tRNA in mass, most humic and fulvic acids possess lower molecular weights than rRNA and mRNA. Thus, an appropriate gel filtration column could be used to remove most of the co-extracted humic substances from an RNA sample. Although several separation media, including Sephadex G-50 (138), Sephadex G-75 (75, 80), Sephacryl S-400 in a MicroSpin S-400 HR column (GE Healthcare, Little Chalfont, UK) (139), and Sepharose CL-6B (106), have been proven to be useful for removing humic substances, we found Sephacryl S-400 to be a better choice with a good balance between the efficient removal of humic substances and the recovery of RNA (139). As the content of carboxyl groups in humic acids increases with a decrease in molecular weight (107), humic acid molecules with a high content of carboxyl groups could be removed more efficiently than other humic acid molecules by cations of various compounds, such as the cetrimonium cation of CTAB and the guanidinium cation of guanidine-containing compounds (3, 43, 44). Because the surfaces of soil humic acids are normally negatively charged (14), the separation of RNA from humic acids can be performed successfully on an ion-exchange column, like Q-Sepharose Fast Flow (76) or a silica-gel-based membrane column (Qiagen Total Nucleic Acid purification system) (54). Apparently, the column purification methods (both gel filtration and ion-exchange columns) are much easier to use and require much less operation time than chemical methods, such as co-precipitation with guanidine hydrochloride followed by phenol extraction (44); therefore, they can be expected to be a standard procedure in the protocol of RNA extraction from soil. Because there is no single purification method to remove co-extracted humic substances completely (45), the appropriate combination of several methods is required to obtain high-purity RNA.

### Measurement of humic substances

**Fig. 2:**
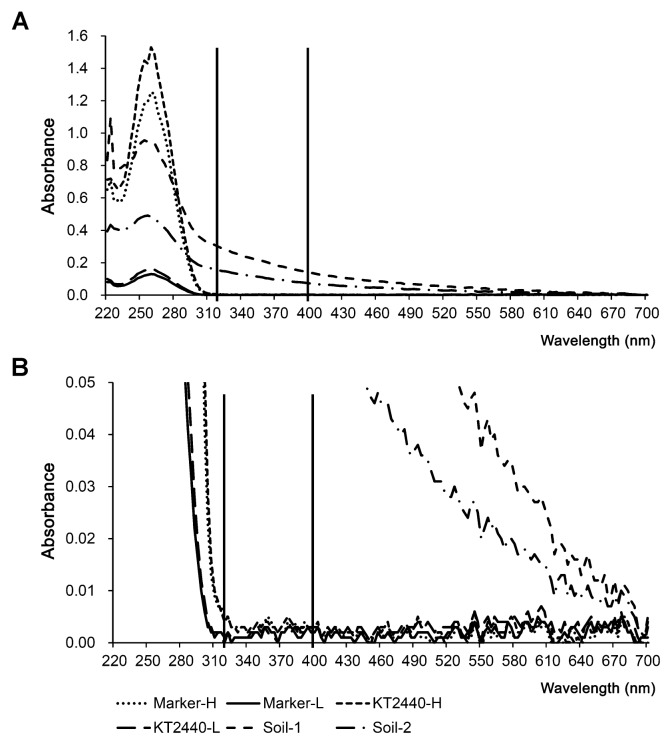
Ultraviolet-visible absorption spectra of RNA samples (A) and the magnified spectra by adjustment of scale (B). Marker-H and Marker-L, Novagen Perfect RNA Marker (0.2–10 Kb) at high (H) or low (L) concentrations; KT2440-H and KT2440-L, a highly purified RNA sample prepared from a pure culture of *Pseudomonas putida* KT2440 strain at high (H) or low (L) concentrations; Soil-1 and Soil-2, two humic-contaminated RNA samples prepared from soil. Vertical lines indicate the positions of absorbance at 320 nm and 400 nm, respectively.

To evaluate the purity of the nucleic acids extracted from soil, several spectroscopic methods for the measurement of co-extracted humic substances were developed, including visual colorimetry (76, 141), visible and ultraviolet (UV) spectroscopy (53, 77, 101, 123), and fluorescence spectroscopy (53, 60, 139). We evaluated these methods and concluded that all of the methods that used visible and UV spectroscopy had the same linear range of measurement with a high tolerance to disturbance by DNA, RNA, and proteins (141); thus, they can serve as routine measurement methods. The methods using fluorescence spectroscopy showed the highest sensitivity to humic acids among all of the methods examined; however, they had low tolerance to disturbance by DNA, RNA, and proteins. Consequently, they can be used in experiments that are especially sensitive to humic acids, such as restriction enzyme digestion, after appropriate dilution of the samples to avoid disturbance by DNA. Although a recent report argued that absorbance at 400 nm was better than that at 320 nm because of RNA disturbance at 320 nm (76), the absorbance of RNA at 320 nm was either the same as the background level (low concentration of RNA) or at an actually negligible level (high concentration of RNA) (Fig. 2), neither of which may affect the measurement of humic acids. Precautions should be taken when measuring the remaining humic acids in a purified RNA sample. The methods for measuring humic acids (visible and UV spectroscopy, and fluorescence spectroscopy) are less sensitive to high than low molecular weight fractions of humic acids because high molecular weight fractions showed lower fluorescent intensity and absorption at the visible and UV regions than low molecular weight fractions (98). Also, high molecular weight fractions are difficult to separate from RNA (138, 139); thus, underestimation of the remaining humic acids in a purified RNA sample may occur.

### Release of RNA from soil

It is known that RNA adsorption by clays decreases with the increase of pH of soil suspensions (40, 121). Adsorption of RNA components, *e.g.*, adenine, adenosine, ribose and adenosine-5′-phosphate (5′-AMP), showed a similar tendency with RNA; in particular, the adsorption of 5′-AMP at pH 4 and pH 6 was about 60 times higher than at pH 8 (46), suggesting that an extraction buffer with a pH higher than 6 could be helpful to release RNA from Andosols. RNA adsorption by allophane increased as the concentration of sodium chloride increased when the pH was higher than 5 (121). Divalent cations, *e.g.*, Ca^2+^ and Mg^2+^, were much more effective at promoting RNA adsorption than mono-cations, *e.g.*, Na^+^ and K^+^(40, 121). Thus, it is preferable for an extraction buffer to possess a pH higher than 6, without Ca^2+^ or Mg^2+^, and with a low level of Na^+^ and K^+^, to improve the recovery efficiency of RNA from Andosols. Although an extraction buffer, which contained sodium phosphate (300 mM, pH 7) but no Mg^2+^ or Ca^+^, has been successfully applied to extract high-purity RNA from a sterilized brown forest soil inoculated with either Gram-positive or -negative bacteria (138, 139), trials using the same extraction buffer failed to extract RNA from Andosols (Wang *et al.*, unpublished data). This result suggests that controlling the pH and divalent cations in an extraction buffer is not sufficient to inhibit the adsorption of RNA by soil; therefore, the optimal buffer composition for RNA release from Andosols should be explored in the future. In successful extractions of DNA from Andosols, an appropriate additive is often required. Some additives have been tested and shown to be helpful in assisting the release of DNA from Andosols to recover DNA from soil (55, 119, 134); however, only one additive, DNA, has been shown to be helpful in recovering RNA from an Andosol (52). In that case, RT-PCR amplification of rRNA was successful, but no functional gene was tested; therefore, it is unclear whether DNA can be helpful in recovering RNA from Andosols for the detection of mRNA. Since many molecules, such as ribose (17, 46, 47), base (17, 18, 46, 47), nucleoside (46), nucleotide (40, 46, 64), DNA (40, 63, 127) and proteins (34, 40), could be adsorbed by soil, it is worth investigating which material is helpful to release RNA from Andosols.

### Removal of rRNA

Removal of rRNA from bacterial total RNA is essential for mRNA enrichment, which is required by some analyses, especially in mRNA sequencing by high-throughput sequencing techniques. To eliminate bacterial rRNA, several methods have been developed: subtractive hybridization with rRNA-specific probes (16, 90, 117); reverse transcription with rRNA-specific primers followed by RNase H digestion to degrade rRNA in rRNA:cDNA hybrids (27); preferential polyadenylation of mRNA (32, 144); recovery of mRNA from gel electrophoresis (73); and digestion with exonuclease that preferentially acts on RNA molecules with a 5′-monophosphate end (including mature rRNA, tRNA, and fragmented mRNA) (95). In a study of RNA-sequencing transcriptomics (also known as RNA-seq) (84, 143) using high-throughput sequencers, subtractive hybridization and exonuclease digestion are popular methods for the removal of rRNA (16, 37, 49, 95, 149). Table 2 lists the commercial kits based on these two methods, although precautions should be taken when using these methods. Because the subtractive hybridization method is based on rRNA-specific probes, the efficiency of rRNA removal will be low if the target rRNA is not compatible with the probes (49). To solve this problem, a sample-specific method was developed and tested by pyrosequencing (116). Also, RNA integrity should be as high as possible when performing subtractive hybridization because more fragmented rRNA molecules have lower removal efficiencies (49, 149). Although the exonuclease digestion method appears to treat any kind of sample, both pure cultures and environmental samples, it has low efficiency for removing Archaea and *Streptomyces* rRNA molecules, possibly because of the special structure of those molecules (49).

### Amplification of RNA

The amount of total RNA prepared for microarray analysis is usually several micrograms to tens of micrograms (87). High-throughput sequencing platforms, such as the Roche 454 FLX Genome Sequencer, Illumina Genome Analyzer, and Applied System SOLiD Sequencer, also require at least 2–5 μg input DNA/cDNA for successful sequencing (147). The amount of RNA required by both techniques is equal to that extracted from 10 to 100 g soil, depending on the soil type and RNA extraction method. If biological replicates are required, the amount of soil for RNA extraction will be much larger. Such large amounts of soil are not always available, and if they are, the cost of preparing RNA samples from large samples is very high. Alternatively, large amounts of RNA can be generated by RNA amplification. The first RNA amplification method was reported by Van Gelder *et al.*(131) in 1990 and it provided the basis of the procedures used today. Ten years ago, this technique was quantitatively evaluated by microarray analysis (5, 102, 132, 137) and modified in many ways, including T7 RNA polymerase-based linear RNA amplification (in vitro transcription, IVT) (79, 83, 151), PCR-based exponential strategy (51, 56), and linear isothermal amplification of cDNA using a single primer (21, 111); however, all of these methods were originally designed for a study using eukaryotic mRNA, which possesses a poly(A) tail at its 3′-end. To amplify prokaryotic RNA, a linear amplification strategy based on T7 RNA polymerase was developed (61, 96). This method uses the overhang tailing activity of the Moloney murine leukemia virus reverse transcriptase to add additional non-template residues, normally cytosines, to the 3′-end of the first strand of cDNA. Then, a T7-promoter-linked oligo(G) primer anneals with these cytosines and primes the synthesis of the second strand of cDNA. Another strategy using a random primer linked to the T7 promoter has been developed (36, 81). In this strategy, T7 promoter sequences are linked to random primers, which are used for the conversion of RNA into cDNA, which, in turn, serves as a template for in vitro transcription to produce amplified RNA. In both strategies, polyadenylation is not necessary. The third strategy for prokaryotic RNA amplification is adding a polyadenylation step prior to IVT. Prokaryotic mRNA with a poly(A) tail at its 3′-end could be amplified by IVT, as with eukaryotic mRNA (31, 136). Cao *et al.*(12) compared three methods: direct labeling with large amounts of total RNA; polyadenylation prior to oligo-dT-primed IVT; and random primed IVT. They found that the method performing polyadenylation prior to oligo-dT-primed IVT is the best choice. Waddell *et al.*(136) also proved that a MessageAmp II-Bacteria Kit (Ambion), which includes a polyadenylation step prior to oligo-dT-primed IVT, is better than random primed IVT. Recently, the MessageAmp II-Bacteria Kit was evaluated by microarray analysis, which showed rather high reproducibility (*r*^2^ between 0.94 and 0.99 for biological replicates) and high fidelity (*r*^2^ between 0.85 and 0.92 for the comparison between amplified RNA and unamplified RNA) (32). Successful applications using this kit in transcriptomic studies using both microarray (142) and high-throughput sequencing (32, 72, 116) platforms have been reported; however, to avoid any bias generated during RNA amplification, Wang *et al.*(142) summarized a common gene list from microarray analyses using both amplified and unamplified RNA for downstream analysis. Although this may risk losing some changed genes, the common gene list definitely contains reliable data.

## Considerations on complexity of RNA extraction from soil

### Cell lysis

To perform comprehensive identification and quantification of microbial transcriptomes by genome-wide unbiased methods, such as genomic tilling array and RNA-seq, it is necessary to prepare high-quality RNA, which should be highly pure, not degraded, and should contain all RNA species in natural proportions (69). Keeping all RNA species in natural proportions is difficult, especially for RNA extracted from soil. Soil contains numerous bacteria of many species, and therefore bias can be easily generated during cell lysis (33). Chemical or enzymatic lysis is relatively gentle and is preferable to lyse Gram-negative bacteria, whereas cell disruption with mechanical methods usually creates a more uniform lysate and disperses soil to allow penetration of the lysis buffer (97). Bead beating, as a mechanical disruption method, can be a better choice for controlling the extraction bias to a low level because of its power to disrupt Gram-positive bacterial cells and spores (33, 58) and its ease of use. In our lab, this method was successfully used to disrupt both Gram-negative (139, 142) and -positive (85, 138) bacteria seeded in soil.

### RNA electrophoresis

After RNA extraction from soil, it is common to run agarose gel electrophoresis to examine the quality of RNA. Under natural conditions, RNA molecules with high GC content can form stable secondary structures, which cause them to move slowly in a gel; therefore, abnormal band patterns can be observed when running an agarose gel using RNA extracted from a GC-rich species (138). To obtain a normal band pattern, denaturation of RNA samples at 70°C for several minutes followed by rapid cooling on ice is necessary (138). RNase inhibitor proteins are often used in RNA extraction protocols to inhibit the activity of RNase; however, many commercial products of RNase inhibitor proteins are inactivated at 70°C so that they cannot protect RNA at such a high temperature. Therefore, to avoid degradation by RNase, the extracted RNA should be highly purified prior to heat denaturation, otherwise a heat-stable RNase inhibitor protein, for example, the RNasin Plus Ribonuclease Inhibitor (Promega, Madison, WI, USA), should be used to inhibit the activity of RNase.

### RNA preparation for different analytical techniques

For RT-PCR-based techniques, such as qRT-PCR and RT-PCR DGGE, a small amount of total RNA, usually from 1 or <1 g soil, is sufficient for the successful detection of target mRNA or rRNA. In such cases, humic substances can be easily controlled to low levels by simple purification. Therefore, the protocol of RNA extraction can be simple and the scale of extraction can be smaller than 1 g soil, e.g., 0.5 g soil per extraction, as used in some reports (3, 43, 67, 76). However, for microarrays and high-throughput sequencing techniques, both of which require large amounts of RNA, pooling of RNA from many extractions is necessary. To reduce the volume of RNA samples to an appropriate size prior to application for these analytical platforms, a concentration procedure is inevitable. During concentration, a low to high level of humic substances can accumulate and interfere with downstream enzymatic reactions; therefore, purification should be performed sufficiently before the concentration procedure (139). To avoid laborious RNA extraction from soil and high costs for many purification columns, whole transcriptome RNA amplification is an alternative method of obtaining large amounts of RNA (142). Although several amplification methods have been reported (36, 61, 81, 96), it is more convenient to use a commercially available kit. To the best of our knowledge, until now, only one commercially available kit has been designed for the amplification of prokaryotic RNA, the MessageAmp II-Bacteria Kit (Ambion). Fortunately, this kit has been evaluated and successfully applied to transcriptomic study. Thus, this RNA amplification kit can be used for soil metatranscriptomic studies.

## Limitations of RNA-based techniques

Although the detection of target mRNA can provide much information about gene function and cell response to treatments or environmental conditions, there are some limitations to the use of RNA-based techniques. First, proteins are molecules that exert gene functions but not mRNA. Thus, detection of target proteins, if possible, should give us more reliable information than the detection of target mRNA. Second, the level of some proteins might not always be consistent with that of the corresponding mRNA. This often happens in Eukarya and Archaea and may also happen in some bacteria, especially high GC Gram-positive species, because of the existence of the proteasome-dependent protein degradation mechanism (6, 38, 39). Third, enzyme proteins usually have a range of optimal conditions to exert activities. Although there is no difference at the mRNA and protein levels, the activity of enzymes may be different among samples because of differences in pH or the existence of activators or inhibitors, as suggested in a recent report (66); therefore, precautions should be taken when explaining the gene expression data obtained from soil samples. If possible, integrating multiple ‘omics’ analyses, including genomics, transcriptomics, proteomics, interactomics, metabolomics, and fluxomics, for soil microbiological study can be a powerful and more reliable method (109, 110, 150).

## Perspectives

### Limited information on methods of RNA extraction from soil

Soil characteristics are important for RNA extraction because RNA extraction can be performed easily using diverse types of soil except for Andosols, and the RNA yields vary among soil types, although they also vary among methods. Unfortunately, such information is not complete in many reports. To avoid redundant studies, we suggest that once a new method of RNA extraction from soil is reported, the following minimum information about the soil used for RNA extraction should be presented, such as soil classification (*e.g.* FAO classification), soil texture, pH, water content, moist soil color (the Munsell color system is preferred), organic and inorganic components. In addition to information about soil characteristics, information about the extracted RNA, such as the UV spectrum, and the ratio of OD_260 nm_/OD_280 nm_ and OD_260 nm_/OD_230 nm_, and the absorbance at 320 nm or other evaluated wavelengths for measurement of humic substances (141) should also be presented to evaluate the purity of the extracted RNA. As a standard of RNA integrity, the data acquired from analysis on an Agilent 2100 Bioanalyzer (Agilent Technologies, Santa Clara, CA, USA) or similar instrument, such as the Experion Automated Electrophoresis System (Bio-Rad Laboratories, Hercules, CA, USA) and MCE-202 MultiNA (Shimadzu Corporation, Kyoto, Japan), should be presented or, alternatively, an image of the agarose gel electrophoresis of RNA samples should be presented.

### Toward a universal method

Until now, no method of RNA extraction from soil could be used to fit all purposes. Thus, significant efforts are still required to develop a universal method, which is expected to facilitate researchers in generating comparable data worldwide. As stated in the motto of the Zymo Research Corporation (Irvine, CA, USA), “The beauty of science is to make things simple.” Thus, we believe that such a universal method should be as simple as possible so that it can be mastered by regular researchers without much experience with RNA experiments. To reach this goal, first, the removal of humic substances should be as simple as possible without loss of purification power. Because biologists have already spent two decades improving the methodology of RNA extraction from soil, it is apparently difficult to simplify the purification procedures based on the current technologies. This may require contributions from chemists or physicists to develop new technologies. Second, RNA extraction from diverse soil types collected worldwide is required. Because it is difficult to conduct such a systematic test for technical, economic, and political reasons, a sub-universal method could be developed, *e.g.*, a method for all soil types in one or several countries. After several sub-universal methods are developed, it may be possible to integrate these methods into an almost universal method.

Gene expression as an important tool in the study of soil microbial ecology and physiology can be expected to be more popular and more important with the development of methods of bacterial RNA extraction from soil.

## Figures and Tables

**Table 1:** Commercially available kits for RNA extraction from soil

Kit	Manufacturer	Soil for processing	Lysis	Purification	Principle of purification
E.Z.N.A. Soil RNA Kit	Omega Bio-Tek (Norcross, GA, USA)	2 g	Bead beating	Single spin column	Adsorption
FastRNA Pro Soil-Direct Kit	MP-Biomedicals (Q-Biogene) (Solon, OH, USA)	0.5 g	Bead beating	Binding matrix	Adsorption
ISOIL for RNA	NIPPON GENE (Tokyo, Japan)	0.5 g	Bead beating	Precipitation	Information not publicly available
IT 1-2-3 Platinum PathTM Sample Purification kit	Idaho Technology (Salt Lake City, UT, USA)	0.5 g	Bead beating	Magnetic beads	Information not publicly available
RNA PowerSoil Total RNA Isolation Kit	MO BIO (Carlsbad, CA, USA)	2 g	Bead beating	Single gravity flow column	Adsorption
Soil Total RNA Purification Kit	Norgen (Thorold, ON, Canada)	0.5 g	Bead beating	Single spin column	Adsorption
ZR Soil/Fecal RNA MicroPrep	Zymo Research (Orange, CA, USA)	0.25 g	Bead beating	Multiple spin columns	Adsorption/gel filtration

**Table 2:** Commercially available kits for rRNA removal

Kit	Principle	Manufacturer	References
MICROExpress Bacterial mRNA Purification Kit	Subtractive hybridization	Ambion (Austin, TX, USA)	(37, 49, 95, 108, 149)
RiboMinus Transcriptome Isolation Kit	Subtractive hybridization	Invitrogen (Carlsbad, CA, USA)	(16)
Ribo-Zero rRNA Removal Kit	Subtractive hybridization	EPICENTRE Biotechnologies (Madison, WI, USA)	(112)
mRNA-ONLY Prokaryotic mRNA Isolation Kit	Exonuclease digestion	EPICENTRE Biotechnologies (Madison, WI, USA)	(49)
